# The Relationship of Tinnitus Distress With Personality Traits: A Systematic Review

**DOI:** 10.3389/fneur.2020.00225

**Published:** 2020-05-29

**Authors:** Juliëtte J. C. M. van Munster, Wouter H. van der Valk, Inge Stegeman, Arno F. Lieftink, Adriana L. Smit

**Affiliations:** ^1^Department of Otorhinolaryngology and Head & Neck Surgery, Leiden University Medical Center, Leiden University, Leiden, Netherlands; ^2^Department of Neurosurgery, Leiden University Medical Center, Leiden University, Leiden, Netherlands; ^3^Department of Otorhinolaryngology and Head & Neck Surgery, University Medical Center Utrecht, Utrecht, Netherlands; ^4^Brain Center Rudolf Magnus, University Medical Center Utrecht, Utrecht, Netherlands

**Keywords:** tinnitus, tinnitus distress, personality traits, five-factor model, neuroticism

## Abstract

**Objectives:** An association between tinnitus distress with anxiety and depression is described in literature. A similar relationship might exist between tinnitus distress and personality traits, especially since associations between personality traits and other chronic diseases are already revealed. In this systematic review, we aim to investigate whether personality is a risk factor for tinnitus distress.

**Design:** We searched PubMed and EMBASE databases from inception up to December 31, 2018 for articles on the association between tinnitus distress and personality. Two researchers screened titles, abstracts, and full texts for eligibility. Directness of evidence and risk of bias were assessed. From the included studies, study characteristics and outcome data of tinnitus distress and personality traits were extracted.

**Results:** A total of 323 unique articles were screened of which 11 cross-sectional studies were eligible for critical appraisal and were used for data extraction. Including study populations were heterogenous, and studies scored high to moderate risk of bias. Nine out of 11 articles showed an association between tinnitus distress and the personality of neuroticism.

**Conclusions:** By limitations in the methodology of included studies, the evidence on specific personality traits as a risk factor for tinnitus distress is inconclusive. Some evidence on a positive association with neuroticism is identified. To draw conclusions about causal relations, these further studies should be of longitudinal design in a cohort setting. These studies should assess tinnitus distress using validated questionnaires with multiple personality dimensions and validated questionnaires to assess personality traits.

## Introduction

Tinnitus is the presence of a buzzing or tinkling sound in one or both ears without an acoustic external source to the head and can be associated with a reduction in health-related quality of life (1–3). Growing evidence shows that many cases of tinnitus are associated with de-afferentation of the central auditory structures due to aging, noise exposure, otologic injury, or other causes. The prevalence of tinnitus varies widely, depending on the used methodology, selected population, and definition of tinnitus. For that reason, the prevalence of the adult population experiencing tinnitus is estimated to be 10–25% (4). Where only 1–7% of the adult tinnitus population seems to struggle with bothersome tinnitus, others experience this symptom without any difficulties, which underlines the heterogeneity of the experienced disease (4). This heterogeneity is not only the experienced diversity in the tinnitus itself (e.g., lateralization, temporal course, sound characteristics) but also due to differences in tinnitus-related problems such as sleep and concentration difficulties and accompanying co-morbidities. Tinnitus-related distress is diverse and can be described as a complex phenomenon associated with negative appraisal, selective attention, safety behaviors, beliefs, and a distorted perception of tinnitus (5, 6). Thereby, it is not surprising that the individual needs of patients for tinnitus-related healthcare are various (4, 7).

So far, it is unclear to what extent psychopathological disorders are predisposing to tinnitus complaints, do aggravate symptoms, or refrain patients from tinnitus relief by diverse tinnitus therapies (8–10). The same might be argued for possible influences of personality traits (10) whereby personality can be defined as the characteristic pattern of thinking, feeling, and behaving of an individual (11). Early appearance of personality after birth indicates that personality is partly genetic determined (12, 13). Many studies have investigated the association between personality and diseases, for instance, between personality and the development of cardiovascular disease and cancer (14–17). A similar relationship could exist between personality characteristics and tinnitus. In the cognitive tinnitus model, it is assumed that tinnitus provokes distress in persons who hold overly negatively thoughts about it, thereby motivating maintaining factors. Subsequently, patients gain a distorted perception of their tinnitus. In 2014, Mucci et al. analyzed the relationship between tinnitus and personality by reviewing the literature from 1968 to 2012 (5, 18). They concluded that tinnitus is associated with several personality characteristics, especially on neuroticism, though a clear distinction between tinnitus perception and tinnitus distress was not made and the quality of data was not assessed. Personality characteristics in patients with more distress might differ from others without high distress levels. A scoping review by Durai et al. in 2016 found associations between tinnitus distress and multiple personality traits such as high neuroticism and low extraversion (19). Still, this review has important limitations due to the scoping method: relevant studies may not have been identified, and a quality assessment was not performed.

To measure personality traits, a wide range of questionnaires can be used. Some questionnaires focus on specific personality traits (19) where other questionnaires are based on models dividing personality into dimensions, for instance, three (Eysenck's theory) or five dimensions [the five-factor model (FFM): the “Big Five”] (19, 20). The variance between factors in the FFM is substantial compared to the variance of other personality inventories (21), and thereby, the FFM adopted a prominent position in the field of personality assessment (22). Assessing personality traits by a validated instrument in tinnitus patients might contribute to a better understanding about the relationship between both. As differences in personality traits can influence tinnitus-related distress and the response on different tinnitus treatments, which is especially important for tinnitus patients with severe complaints of tinnitus, this insight might be of benefit to understand the disease and treatment outcome. Therefore, we aim to investigate whether personality is a risk factor for tinnitus distress by a systematic review of the literature.

## Materials and Methods

### Search Strategy

We searched PubMed and Embase to access records, which analyzed the relationship between tinnitus distress and personality up to December 2018. PubMed was searched by entering the following search criteria in PubMed: [personal^*^(Title/Abstract)] AND {[tinnitus (MeSH Terms)] OR tinnitus (Title/Abstract)}. A similar search strategy was used to search Embase. The Preferred Reporting Items for Systematic Reviews and Meta-analysis (PRISMA) was used as a writing guideline (23).

### Study Selection

After removal of duplicates, two researchers (JM and WV) independently screened titles and abstracts of searched articles. Eligible full text articles were retrieved through the databases and by emailing the authors. Full text eligibility was determined by the in- and exclusion criteria listed in Table 1. We included studies if they included adults with tinnitus that correlated tinnitus distress with personality characteristics. With tinnitus distress, terms such as tinnitus loudness, annoyance, and handicap were also included. When there was a different opinion on eligibility, the two reviewers tried to reach consensus. Studies investigating a therapy for tinnitus were excluded since interventions can change personality traits (24). The third and fifth author (IS and AS) were consulted if no unanimity was achieved.

**Table 1:** Eligibility criteria.

Inclusion criteria
Studies that correlated tinnitus distress with personality characteristics
Participants ≥18 years of age
Exclusion criteria
Studies that correlated tinnitus with psychiatric disease or pathological personality
Studies on personality characteristics in patients with hearing loss, dizziness, or chronic disease
Validation studies of a questionnaire
Reviews or case reports
Studies investigating a therapy for tinnitus

### Data Collection

Two researchers (JM and WV) extracted data regarding study design, study population, group size, setting, questionnaires regarding tinnitus distress and personality, and outcomes of the evaluation concerning tinnitus distress and personality. Primary outcome was the relationship between tinnitus distress and personality traits, as described by the FFM. The FFM is a taxonomy of five traits: neuroticism, extraversion, openness, agreeableness, and conscientiousness (25). The Big Five Personality Dimensions Scale, the NEO Five-Factor Inventory (NEO-FFI), and the Big Five Inventory (BFI) are based on the FFM. When included trials used personality trait questionnaires not based on the FFM categorization, these personality traits were matched to the FFM categories. For this, the literature was searched to find a correlation between the non-FFM personality trait category and the traits as defined and scored by the FFM (26–34). Those associations and stratifications between FFM personality traits and non-FFM traits are described in Table 2. Mean scores of the questionnaires including standard deviations and *P*-values were distracted from the articles when available. When a *t-*test was used, the *F*-ratio and *P*-value were documented.

**Table 2:** Correlation between non-FFM personality questionnaires and the FFM personality traits based on the literature.

**Questionnaire**	**FFM neuroticism**	**FFM extraversion**	**FFM openness**	**FFM agreeableness**	**FFM conscientiousness**	**References**
EPI-Neuroticism	Neuroticism	–	–	–	–	(35)
EPQ	Neuroticism	Extraversion	–	Psychoticism (R)	Psychoticism (R)	(35)
FMPS	Mistakes, standards, parent criticism	–	Parent criticism, doubts	Parent expectations (R)	Standards (R), doubts, organization	(33)
FPI-R	Life satisfaction (R) Excitability Strain Somatic complaints Emotionality	Inhibitedness (R) Extraversion Achievement orientation Aggressiveness	Openness	Social orientation Aggressiveness (R)	Need for achievement	(36)
MMPI	Hypochondriasis Depression Psychasthenia	Social introversion (R)	Masculine/feminine (R)	Paranoia (R)	Psychopathic deviate (R)	(37)
MPQ	PEM (R), NEM	PEM (R)	Constraint (R)	NEM (R)	PEM, constraint	(26)
TCI	Harm avoidance Self-directedness (R)	Harm avoidance (R) Persistence	Self-transcendence	Cooperativeness Reward Dependence	Self-directedness Persistence Novelty Seeking (R)	(28, 31)

*Different personality questionnaires and their domains were compared and correlated to the personality traits of the FFM. Reverse correlations are depicted by (R). EPI, Eysenck Personality Inventory; EPQ, Eysenck Personality Questionnaire; FMPS, Frost Multidimensional Perfectionism Scale; FPI-R, Freiburger Personlichkeitsinventar; MMPI, Minnesota Multiphasic Personality Inventory; MPQ, Multidimensional Personality Questionnaire; NEM, Negative Emotionality superfactor; TCI, Temperament and Character Inventory; PEM, Positive Emotionality superfactor*.

### Risk of Bias

Critical appraisal of selected studies regarding risk of bias (RoB) was performed by two researchers (WV and JM), using the Newcastle–Ottawa Quality Assessment Scale, using different assessment scales for cohort studies and for case-control studies (38). This instrument is divided into three subdomains concerning the bias in selection, comparability, and exposure. Each subdomain consists of one to four categories of potential bias. A study can be awarded one or two stars depending on each item within the categories. Selection can be awarded with a maximum of four stars, comparability with a maximum of two stars, and outcome with a maximum of three stars. The more stars were earned, the lower the risk of bias of the study was rated.

## Results

### Study Selection

**Figure 1:**
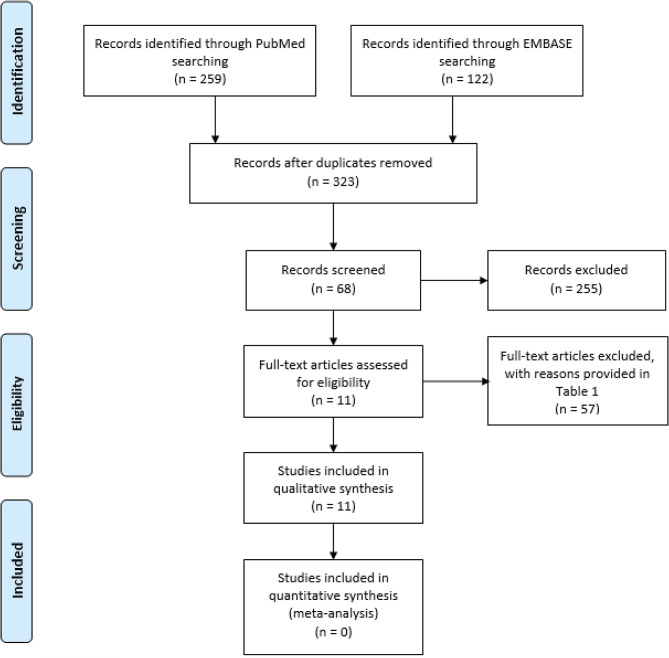
The Preferred Reporting Items for Systematic Reviews and Meta-Analyses (PRISMA) Flow Diagram of Study Selection (39).

Our flow chart of the study selection, including reasons for exclusion of articles, is shown in Figure 1. After removal of duplicates, 323 unique records were identified. Of these, 255 articles were excluded based on title and abstract screening. After reading the full text of 68 articles, 11 articles were eligible for critical appraisal.

### Risk of Bias and Quality Assessment

Critical appraisal of the 11 articles is outlined in Table 3. None of the studies were awarded with the maximal amount of nine stars; the range is between three and six stars. The majority used a selected group of tinnitus patients by including them from outpatient clinics (40, 41, 43, 44, 46–49). Furthermore, none of the case-control studies did provide information about non-respondents, and all cohort studies were of cross-sectional study design hindering the assessment of adequate follow-up. In addition, the majority of studies did not correctly control for confounding factors (40, 41, 43–49). Together, this limits the number of stars awarded for all subdomains. Only two studies used a representative cohort of patients with tinnitus out of the general population; however, these studies used a single question of tinnitus distress in contrast to validated multi-item questionnaires (45, 50).

**Table 3:** Risk of bias assessment using the Newcastle–Ottawa Quality Assessment Scale.

**References**	Selection (Max. ****)	Comparability (Max. **)	Outcome (Max. ***)	**Total number of stars^**b**^**
Adami Dehkordi et al. (40)^a^	***		**	*****
Andersson et al. (41)	**		*	***
Durai et al. (42)^a^	***	**	*	******
Gerber et al. (43)	*		**	***
Langguth et al. (44)	**		*	***
McCormack et al. (45)	***			***
Meric et al. (46)	**	**	*	*****
Salviati et al. (47)	**		*	***
Strumila et al. (48)	**		*	***
Weber et al. (49)	**		*	***
Welch and Dawes (50)	***	**		*****

a *Newcastle–Ottawa Quality Assessment Scale for case-control studies was used*.

b *A maximum of nine stars could be earned in total and implies the lowest Risk of Bias*.

#### Characteristics of Studies

There was a large heterogeneity in tinnitus questionnaires and personality questionnaires to measure tinnitus distress and personality traits, respectively, in the included studies. Because of this heterogeneity, no meta-analysis could be performed. Therefore, a descriptive analysis of the included articles is provided. All but three (42, 45, 50) studies included patients who visited the tinnitus department or Ear-, Nose-, Throat (ENT), which leads to a specific patient selection. The two cohort studies included patients from large-population studies in Dunedin (New Zealand) and the United Kingdom (UK) (45, 50) and assessed personality traits in a cross-sectional manner.

### Synthesis of the Results

Extensive descriptions of the articles and outcomes are presented in Table 4.

**Table 4:** Summary of studies on personality characteristics in tinnitus patients according to tinnitus distress.

**References**	**Questionnaires (personality, tinnitus)**	**Study design**	**Study population**	**Sample number (Tinnitus number)**	**Outcomes**
Andersson et al. (41)	HADS, ISI, FMPS TRQ	Case series Cross-sectional	Tinnitus patients visiting the Audiology Department	256 (256)	Severity of tinnitus was correlated with Concern over Mistakes, Personal Standards, Parental Expectations, Parental Criticism, Doubts about Action and Organization (*p* < 0.05)
Adami Dehkordi et al. (40)	EPQ TET, TSI	Case control Cross-sectional	Patients with idiopathic tinnitus from the ENT Department and matched, healthy non-tinnitus volunteers from patients' family	66 (33)	No associations between the TSI, the 1–10 Tinnitus Scale Score and any of the personality traits were found
Durai et al. (42)	MPQ TFI	Case control Cross-sectional	Tinnitus sufferers recruited from the University of Auckland Tinnitus Research Volunteer Database Non-tinnitus sufferers recruited via the University of Auckland Hearing and Tinnitus database, posters, and social media	215 (61)	Within tinnitus sufferers, stress reaction scores were negatively correlated with constraint (*r* = −0.365, *p* = 0.004) and social closeness/positive emotionality (*r* = −0.296, *p* = 0.02) and positively correlated with alienation scores/negative emotionality (*r* = 0.406, *p* = 0.001) Total TFI scores was significantly negatively correlated with social closeness (*r* = −0.312, *p* < 0.001) The tinnitus group had lower levels of self-control scores (mean = 12.70, SD = 4.15) and social closeness scores (mean = 8.46, SD = 3.57) than controls (respectively, mean = 13.76, SD = 3.26; mean = 10.11, SD = 4.06) and higher alienation scores (mean = 3.48, SD = 3.87) than controls (mean = 10.11, SD = 4.06)
Gerber et al. (43)	MMPI, scaling method to assess severity of stress events by Homes and Rahe 26-item questionnaire designed to obtain a subjective measure of tinnitus severity (not validated)	Case series Cross-sectional	Male tinnitus patients referred to the Audiology Clinic with subjective tinnitus as a primary complaint	45 (45)	Severity of tinnitus was not related to stress events or differences on the MMPI profile
Langguth et al. (44)	NEO-FFI, BDI THI, TQ	Case series Cross-sectional	Patients with tinnitus for at least 6 months, visiting the Tinnitus Clinic	72 (72)	High tinnitus handicap was correlated with low agreeableness (*p* = 0.003), but not with neuroticism, extraversion, openness, and conscientiousness High tinnitus severity was correlated with high neuroticism (*p* = 0.028), but not with extraversion, openness, agreeableness, and conscientiousness
McCormack et al. (45)	EPI-Neuroticism Single question concerning tinnitus annoyance	Cohort Cross-sectional	Tinnitus, and non-tinnitus participants, aged 40 to 69 years, recruited from the UK Biobank Dataset	172.621 (27.964)	Neuroticism had a stronger association with bothersome tinnitus [4.11 (OR 95% CI 3.69–4.58); *p* < 0.001] than tinnitus presence [OR 2.11 (95% CI 2.00–2.22); *p* < 0.001]
Meric et al. (46)	French short MMPI TRQ, THQ, STSS scale	Case series Cross-sectional	Tinnitus patients visiting the ENT Department	281 (281)	Increasing tinnitus impact and handicap were correlated with paranoia, psychasthenia, schizophrenia, and hypochondriasis (*p* < 0.0001). No association was present for mania
Salviati et al. (47)	VRS, SCL90-R, TCI THI	Case series Cross-sectional	Tinnitus patients from the Tinnitus Center of the Department of Sense Organs	239 (239)	Tinnitus handicap was associated with all psychopathological dimensions (somatization, obsessive–compulsive, anxiety, depression, sensitivity, hostility, phobic anxiety, paranoid ideation, psychoticism; *p* < 0.001) Harm avoidance and self-directedness were also correlated with tinnitus handicap (*p* < 0.004), but not with reward dependence (*p* = 0.88), novelty seeking (*p* = 0.90), persistence (*p* = 0.70), cooperativeness (*p* = 0.32), and self-transcendence (*p* = 0.21)
Strumila et al. (48)	HADS, short version of Big Five Personality Dimensions Scale and sociodemographic questions THI, VAS of tinnitus severity	Case series Cross-sectional	Tinnitus sufferer recruited via internet	212 (212)	Tinnitus severity was not correlated with personality traits [in mean (SD)]: - Extraversion mean 26.49 (5.13) - Agreeableness 26.46 (5.13) - Consciousness 26.33 (4.02) - Neuroticism 18.55 (5.45) - Openness 21.39 (5.32)
Weber et al. (49)	FPI-R, BDI TQ	Case series Cross-sectional	Adult, tinnitus patients visiting the ENT Department	121 (121)	Based on tinnitus severity, patients differed in - Life satisfaction (F = 5.497; *p =* 0.001), lower if increasing severity - Excitability (F = 5.151; *p =* 0.002), higher if increasing severity - Aggressiveness (F = 3.483; *p =* 0.018), higher if increasing severity - Stress (F = 10.846; *p* < 0.001), higher if increasing severity - Physical complaints (F = 12.876; *p* < 0.001), higher if increasing severity - Health concerns (F = 3.610; *p =* 0.015), higher if increasing severity - Emotionality (F = 12.708; *p* < 0.001), higher if increasing severity
Welch and Dawes (50)	MPQ Single question concerning tinnitus annoyance	Cohort Cross-sectional	Tinnitus and non-tinnitus participants born between April 1972 and March 1973 from a birth cohort of Dunedin (New Zealand's South Island)	970 (437)	Based on amount of tinnitus, personality differed significantly: Positive emotionality [F_(2.946)_ = 3.675; *p =* 0.026] - Social closeness lower in groups with more tinnitus [*F*_(2.946)_ = 13.242; *p* < 0.001] - Well-being, social potency, and achievement did not differ Negative emotionality [*F*_(2.946)_ = 12.810; *p* < 0.001] - Stress reaction [*F*_(2.946)_ = 9.144, *p* < 0.001] higher in groups with more tinnitus - Alienation higher in groups with more tinnitus (Wald = 12.206; p <0.001) - Aggression did not differ Constraint [*F*_(2.946)_ = 5.516; *p =* 0.004] - Self-control lower in groups with more tinnitus [*F(2.946)* = 4.752; *p =* 0.009] - Harm avoidance or Traditionalism did not differ Based on the annoyance of tinnitus, personality differed significantly: Positive emotionality [*F*_(2.424)_ = 3.010, *p =* 0.050] - Social closeness lower in patients more tinnitus distress [*F*_(2.424)_ = 3.471; *p =* 0.032] - Well-being lower in groups with more annoyance (Wald = 6.579, *p =* 0.010) - Social potency or achievement did not differ Negative emotionality [*F*_(2, 424)_ = 6.039, *p =* 0.003) higher in groups with more annoyance. Annoyance was not related to constraint [*F*_(2.424)_ = 1.722, *p =* 0.180].

*Secondary outcome on tinnitus distress, annoyance, or severity on personality characteristics in tinnitus patients per publication (first author, year of publication). Per publication, the used tinnitus and personality questionnaires used are depicted; if underlined, they assessed personality traits. Study design and information on the included populations are shown. The outcomes show results of tinnitus distress in relation to personality traits in tinnitus patients. BDI, Beck Depression Inventory; EPI, Eysenck Personality Inventory; EPQ, Eysenck Personality Questionnaire; FMPS, Frost Multidimensional Perfectionism Scale; FPI-R, Freiburger Personlichkeitsinventar; HADS, Hospital Anxiety and Depression Scale; ISI, Insomnia Severity Index; MMPI, Minnesota Multiphasic Personality Inventory; MPQ, Multidimensional Personality Questionnaire; NEO-FFI, NEO Five-Factor Inventory; SCL-90-R, Symptom Checklist-90-Revised; STSS, Subjective Tinnitus Severity Scale; TCI, Temperament and Character Inventory; TET, Tinnitus Evaluation Test; TFI, Tinnitus Functional Index; THI, Tinnitus Handicap Inventory; THQ, Tinnitus Handicap Questionnaire; TQ, Tinnitus Questionnaire; TRQ, Tinnitus Reaction Questionnaire; TSI, Tinnitus Severity Index; VAS, Visual Analog Scale; VRS, Stress-Related Vulnerability Scale*.

#### Tinnitus Distress and Personality Traits

A majority (9 out of 11 studies) of the included studies showed one or more associations (both positive and negative) between tinnitus distress and personality (41, 44–47, 49, 50). Two out of 11 articles studied the association between tinnitus distress and the FFM personality traits by usage of the NEO five-factor inventory (44) or a short version of the Big Five Personality dimensions scale (48). Of those, Langguth et al. did find a negative association between scores on the Tinnitus Handicap Inventory (THI) and consciousness (correlation coefficient = −0.367, *p* = 0.003), and a positive association between tinnitus distress on the Tinnitus Questionnaire (TQ) and neuroticism (correlation coefficient = 0.276, *p* = 0.028) (44). Furthermore Strumila et al. (48) stated that high THI scores did not predict high scores on any of the personality traits, but neuroticism did influence distress of tinnitus perception (*p* <0.001, standardized coefficient 0.38).

The other nine studies used non-FFM-based questionnaires to assess personality traits in similar domains to the FFM division (40–43, 45–47, 49, 50). To be able to make a comparison, we converted these outcomes of personality traits to the FFM personality traits when a comparison between both was described in literature, as stated previously (Tables 2, 5). One study found a positive association between bothersome tinnitus and neuroticism [odds ratio (95% CI) = 4.11 (3.69–4.58), *p* < 0.001] using the EPI-neuroticism questionnaire (45). Eight of nine non-FFM-based studies indicated a positive correlation between neuroticism and increased tinnitus distress, including the New Zealand study, which represents a sample of the general population (Table 5) (40–42, 45–47, 49, 50). For the other categories of personality traits (extraversion, openness, agreeableness, and consciousness), we found no conclusive results about an association with tinnitus distress.

**Table 5:** Association of personality trait outcomes to tinnitus distress.

**FFM**	**Association between tinnitus distress and personality**		
	**Positive association**	**Negative association**	**No association found**
Neuroticism	Neuroticism (44)^*^(45) Positive emotionality (42, 50) Negative emotionality (42) Parental criticism (41) Concerns over mistakes (41) Hypochondriasis (46) Depression (46) Psychasthenia (46) Harm avoidance (47) Self-directedness (47) Life satisfaction (49) Excitability (49) Strain (49) Somatic complaints (49) Emotionality (49)		Neuroticism (48)^*^ Neuroticism (40) Hypochondria (43) Depression (43) Psychasthenia (43)
Extraversion	Aggressiveness (49)	Social introversion (46) Social closeness (50) Positive emotionality (42, 50)	Extraversion (44, 48)^*^ Extraversion (40) Social introversion (43) Wellbeing, social potency, achievement (50)
Openness	Constraint (50) Parental criticism (41) Doubts about Action (41)	Masculinity/Femininity (46)	Openness (44, 48)^*^ Harm avoidance and traditionalism (50) Masculine/feminine (43)
Agreeableness	Parental expectations (41)	Agreeableness (48)^*^ Negative emotionality (42)	Psychoticism (40) Agreeableness (44)^*^ Negative emotionality Paranoia (46) Aggressiveness (49) Paranoia (43)
Consciousness	Personal standards (41) Doubts about action (41)	Consciousness (44, 48)^*^ Constraint (42) Positive emotionality (42) Self-control (50)	Psychoticism (40) Positive emotionality (50) Harm avoidance and traditionalism (50) Psychopathic deviate (43, 46)

* *Personality traits as measured using the FFM personality traits. Other personality traits are “translated” according to Table 2 and clustered under the corresponding FFM personality traits*.

## Discussion

In the present review, we studied personality traits as a risk factor for tinnitus distress, in which 11 cross-sectional studies with a high degree of heterogeneity in methods and outcomes were included. Owing to heterogeneity in the reported personality traits, it was not possible to perform a meta-analysis. Of the FFM, neuroticism was the only trait with a positive relationship with tinnitus distress in multiple studies. The association between neuroticism and tinnitus distress is in line with previous findings about emotional health and tinnitus: tinnitus distress shows close relationships with factors related to emotional health, and emotional health may be prognostic for the development of tinnitus distress (51, 52). One could argue that patients with more severe complaints of tinnitus are indeed more likely to have a neurotic personality, since neuroticism is described as the tendency to experience negative emotions. However, neuroticism is also associated with psychopathological symptoms of depression and anxiety (53), which complicates the causality of this association, since anxiety and depression are more prevalent among tinnitus patients (8, 19, 54). Moreover, both tinnitus and personality are potentially influenced by genetic factors (55, 56). Interestingly, of the FFM traits, the strongest associations were revealed between genetic variants and neuroticism (56–58). One of these studies used the same prospective cohort in the United Kingdom as the study of McCormack et al. (45) of which results were included in this systematic review. Indeed, this study found a positive association between neuroticism and tinnitus.

Several major limitations of this systematic review must be mentioned. Most of the studies included a selected population of tinnitus patients visiting an ENT department, audiological clinic, or tinnitus clinics and, thus, are not necessarily representative of the general population. For instance, headache-prone individuals have found to differ in personality profiles when comparing institutionalized patients to non-institutionalized controls from outside the clinic (59). Potentially, help-seeking tinnitus patients might differ in personality from non-help-seeking tinnitus patients. Owing to the selected sample of tinnitus patients used in the included studies in our review, generalization of outcomes is hindered. Moreover, most studies are prone to bias as scored by the Newcastle–Ottawa Quality Assessment Scale. For instance, confounders in the relation between personality traits and tinnitus distress exist, but not all studies described or analyzed these potential confounding factors such as gender (41, 48, 50). Additionally, as many different questionnaires were used to assess the personality traits, we used previously described correlations to convert the personality domains out of these questionnaires into the FFM categorization, to be able to compare outcomes of different studies. To our knowledge, however, this method is not validated nor described previously.

For all these reasons, the evidence we present in this systematic review about the relationship between personality and tinnitus is inconclusive. The aim of our review was to assess personality as a risk factor for tinnitus distress. Longitudinal cohort studies are needed to assess the causality of personality in tinnitus, address the limitations, and reveal additional potential confounders in this relationship.

The potential relationship of tinnitus distress with neuroticism may affect the success of specific tinnitus treatments, making the execution of high-quality studies on this topic of eminent importance. For example, cognitive behavioral treatment (CBT) targets the emotional reactivity and (dysfunctional) behavioral mechanisms. Studies assessing tinnitus outcome after CBT treatment show a reduction in disease-related distress and increased daily life functioning in individuals with subjective tinnitus (60–62). Interestingly, FFM personality can affect the tinnitus outcome after CBT treatment as demonstrated by Kleinstauber et al. in a clinical trial (63). Therefore, one should not only consider tinnitus distress (64) but also individual characteristics as personality traits to tailor tinnitus treatment for this heterogenous group of patients.

## Conclusion

We endeavored to present the evidence on the relationship between tinnitus distress and personality traits in this systematic review. Owing to limitations in the methodology of included studies, the evidence on specific personality traits as a risk factor for tinnitus distress is inconclusive. Some evidence on a positive association with neuroticism is identified. To draw conclusions about causal relations, future studies should be of longitudinal design in a cohort setting. These studies should assess tinnitus distress using validated questionnaires with multiple personality dimensions and validated questionnaires to assess personality traits.

## Data Availability Statement

The datasets generated for this study are available on request to the corresponding author.

## Author Contributions

This study was conceived and designed by JM, WV, IS, and AS. All authors contributed to acquisition, analysis, and interpretation of data. The drafting of the manuscripts was performed by JM and WV. Critical revision of the manuscript for important intellectual content as well as study supervision was performed by AS, IS, and AL.

## Conflict of Interest

The authors declare that the research was conducted in the absence of any commercial or financial relationships that could be construed as a potential conflict of interest.
